# Rapid and easy construction of a simplified amplicon sequencing (simplified AmpSeq) library for marker-assisted selection

**DOI:** 10.1038/s41598-023-37522-1

**Published:** 2023-06-29

**Authors:** Sogo Nishio, Shigeki Moriya, Miyuki Kunihisa, Yukie Takeuchi, Atsushi Imai, Norio Takada

**Affiliations:** 1 Institute of Fruit Tree and Tea Science, NARO, 2-1 Fujimoto, Tsukuba, Ibaraki 305-8605 Japan; 2 Institute of Fruit Tree and Tea Science, NARO, Morioka, Iwate 020-0123 Japan

**Keywords:** Agricultural genetics, Plant breeding

## Abstract

Marker-assisted selection (MAS) is fundamental for plant breeding programs, as it can identify desirable seedlings at a young stage and reduce the cost, time and space needed for plant maintenance, especially for perennial crops. To facilitate the process of genotyping, which is time consuming and laborious, we developed a simplified amplicon sequencing (simplified AmpSeq) library construction method for next-generation sequencing that can be applied to MAS in breeding programs. The method is based on one-step PCR with a mixture of two primer sets: the first consisting of tailed target primers, the second of primers that contain flow-cell binding sites, indexes and tail sequences complementary to those in the first set. To demonstrate the process of MAS using s implified AmpSeq, we created databases of genotypes for important traits by using cultivar collections including triploid cultivars and segregating seedlings of Japanese pear (*Pyrus pyrifolia* Nakai), Japanese chestnut (*Castanea crenata* Sieb. et Zucc.) and apple (*Malus domestica* Borkh.). Simplified AmpSeq has the advantages of high repeatability, ability to estimate allele number in polyploid species and semi-automatic evaluation using target allele frequencies. Because this method provides high flexibility for designing primer sets and targeting any variant, it will be useful for plant breeding programs.

## Introduction

With advances in molecular genetic technology, marker-assisted selection (MAS) has become fundamental and essential for plant breeding^1,2^. It is increasingly common that molecular markers linked to traits of interest in commercial crops have been identified and that these markers can be practically used in breeding programs^3–5^. Instead of evaluating phenotypic traits in mature plants, a laborious and time-consuming procedure, MAS allows breeders to determine the genotypes for target traits easily and quickly when the plants are young, enabling breeders to reduce the cost of raising plants and the amount of field space required. In a 2014 summary, studies of the Poaceae (including rice, wheat and maize) accounted for 60% of plant MAS publications^3^. On the other hand, MAS has been less widely adopted in perennial crops such as fruit trees^4^, despite the fact that it offers tremendous advantages for these species^6^ because the trees have a long juvenile phase and are large, and the number of trees that can be managed is limited.

Until recently, genotyping for MAS was usually conducted using agarose gel electrophoresis for molecular markers such as sequence-characterized amplified regions (SCAR) and cleaved amplified polymorphic sequences (CAPS) and using capillary electrophoresis for simple sequence repeats (SSRs). Because these procedures generally require visual scoring to determine genotypes, unintentional human errors are inevitable. Moreover, it is labor-intensive to detect and identify each target band for each important trait in studies involving more than a few markers and more than a thousand individuals, as would be needed for MAS in breeding programs. Although SSRs are suitable for MAS owing to their multi-allelic nature, codominance and high transferability across cultivars and species^7^, the existence of slippage and stutter bands makes allele determination difficult^8^. Also, those traditional genotyping methods are not suitable for genotyping for polyploid plants, in which it is necessary to detect and count duplicated alleles. To overcome these difficulties and further improve breeding programs, an easy, fast and flexible method to identify genotypes is required.

With the advent of next-generation sequencing (NGS) technology, the cost and time needed for sequencing has dramatically decreased^9^. Reference genomes have been constructed for most commercial crops, accelerating the development of molecular markers and the identification of genes of interest. Methods such as genotyping by sequencing (GBS), restriction-site-associated DNA sequencing (RAD-seq), genotyping by random amplicon sequencing-direct (GRAS-Di) and multiplexed inter-simple sequence repeat genotyping by sequencing (MIG-seq) have been practically used to construct genetic maps and to conduct genome-wide association studies (GWAS) and population genetics studies^10–14^. These methods are used to sequence specific regions in a genome that are located between restriction enzyme sites or random primer annealing sites and that can be used to identify considerable numbers of single-nucleotide polymorphisms (SNPs) in cultivar collections or breeding populations. On the other hand, advances in NGS technology have had relatively less effect on improvement of MAS. To genotype specific SNPs linked to genes of interest for MAS, an amplicon sequencing method (AmpSeq) has been developed and used in case studies^15,16^. However, AmpSeq requires two-step PCR to add adaptors to be detected by the NGS platform, which takes twice the time and cost of a normal one-step PCR procedure for genotyping. Also, in recent years, Kompetitive Allele-Specific PCR (KASP) has been applied in some breeding programs^17–19^, but it is not suitable for multiplex PCR or for use with crude DNA.

The objectives of this study were (1) to develop a new, simple method for genotyping and allele calling for MAS using a simplified amplicon sequencing process (“simplified AmpSeq”), (2) to create a database for MAS using simplified AmpSeq in Japanese pear, Japanese chestnut and apple and demonstrate that the method is useful for breeding programs, and (3) to confirm the repeatability and versatility of this method, including for analysis of triploid cultivars.

## Results

### Constructing a simplified AmpSeq library for NGS using simplified one-step PCR

**Figure 1:**
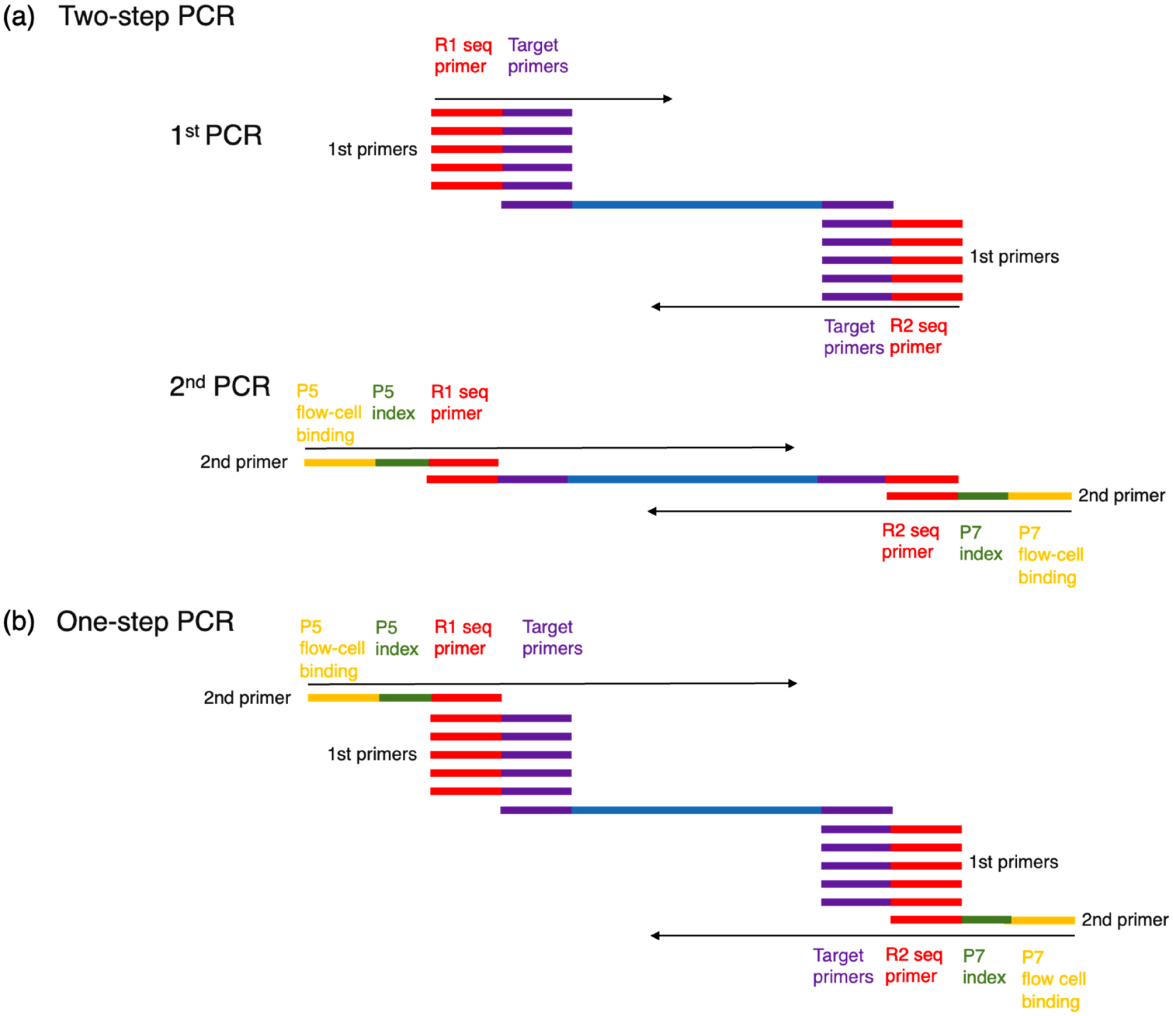
Summary of library construction for next-generation sequencing using (**a**) two-step PCR and (**b**) simplified one-step PCR. A different set of indexes (green) was added to each sample (i.e., cultivar or seedling) to allow the sequences from that sample to be distinguished after multiplexing.

In general, amplicon sequencing libraries for NGS are constructed by two-step PCR: a 1st PCR to amplify target sequences and a 2nd PCR to extend adapters responsible for flow-cell binding and indexes for discriminating individuals (Fig. 1a)^16,20^. To simplify the process of constructing an amplicon library for MAS, the possibility of using simplified one-step PCR, in which the 1st and 2nd primer sets are used in a single reaction, was investigated (Fig. 1b). The 1st primers contained pairs of sequences to amplify the target regions and extended sequences containing R1 and R2 seq primers. The 2nd primers contained the extended sequences along with Illumina flow-cell binding sites and different 8-bp indexes. Each sample (cultivar or seedling) was identified by a unique pair of indexes. By using pear and chestnut cultivars and primers that were already being used in breeding programs (Tables 1 and 2; Supplementary Tables 1 and 2), the possibility of amplifying target bands by simplified one-step PCR was preliminarily confirmed. The bands detected by agarose gel electrophoresis were of the size required for NGS libraries (242–271 bp), which included the target sequences of the markers (107–136 bp) and extended sequences (135 bp) composed of R1 and R2 primers, flow-cell binding sites and indexes.

**Table 1:** Cultivars, selections and seedlings used in this study.

Plant	Sample sizes	Parentage of seedlings
Pear	59 cultivars and selections	
	96 seedlings	48 seedlings derived from crosses between Tsukuba 63 and 515-020
		48 seedlings derived from crosses between Tsukuba 60 and 592-021
Chestnut	46 cultivars and selections	
	24 seedlings	24 seedlings derived from crosses between 776-013 and 794-017
Apple	52 cultivars and selections	
	66 seedlings	66 seedlings derived from crosses between 7-4151 and 8-818

**Table 2:** Markers used in this study.

Marker	Plant	Trait	Type	Chr	Start	End	Size (bp)	Reference No.
TsuENH101	Pear	Pear scab resistance	SSR	1	9,845,756	9,845,882	127	38
TsuENH157.mod		Pear scab resistance	SSR	1	12,536,169	12,536,041	129	38
PPACS2.mod		Fruit-ripening day	SSR	15	12,380,055	12,379,942	114	40
Psc07		Fruit skin color	SSR	8	4,160,692	4,160,574	119	34
Mdo.chr11.34.modA		Pear black spot resistance	SSR	11	2,276,164	2,276,299	136	43
Co	Apple	Columnar type	Dominant marker	10			128	24
CH-Vf1		Apple scab resistance	SSR	1	27,995,071	27,995,204	134	42
MdPG1.mod		Flesh mealiness	SSR	10	27,298,443	27,298,550	108	45
Alt_indel		Apple black spot resistance	Indel	11	2,804,575	2,804,736	162	44
Mdo.chr11.34.modB		Apple black spot resistance	SSR	11	2,824,157	2,824,283	127	43
CmSca06716.mod	Chestnut	Pellicle peelability	SSR	A	12,150,467	12,150,575	109	23
CCR1.0F_56177061		Pellicle peelability	SNP	F	56,177,032	56,177,138	107	41

### Determining optimal primer concentrations

**Figure 2:**
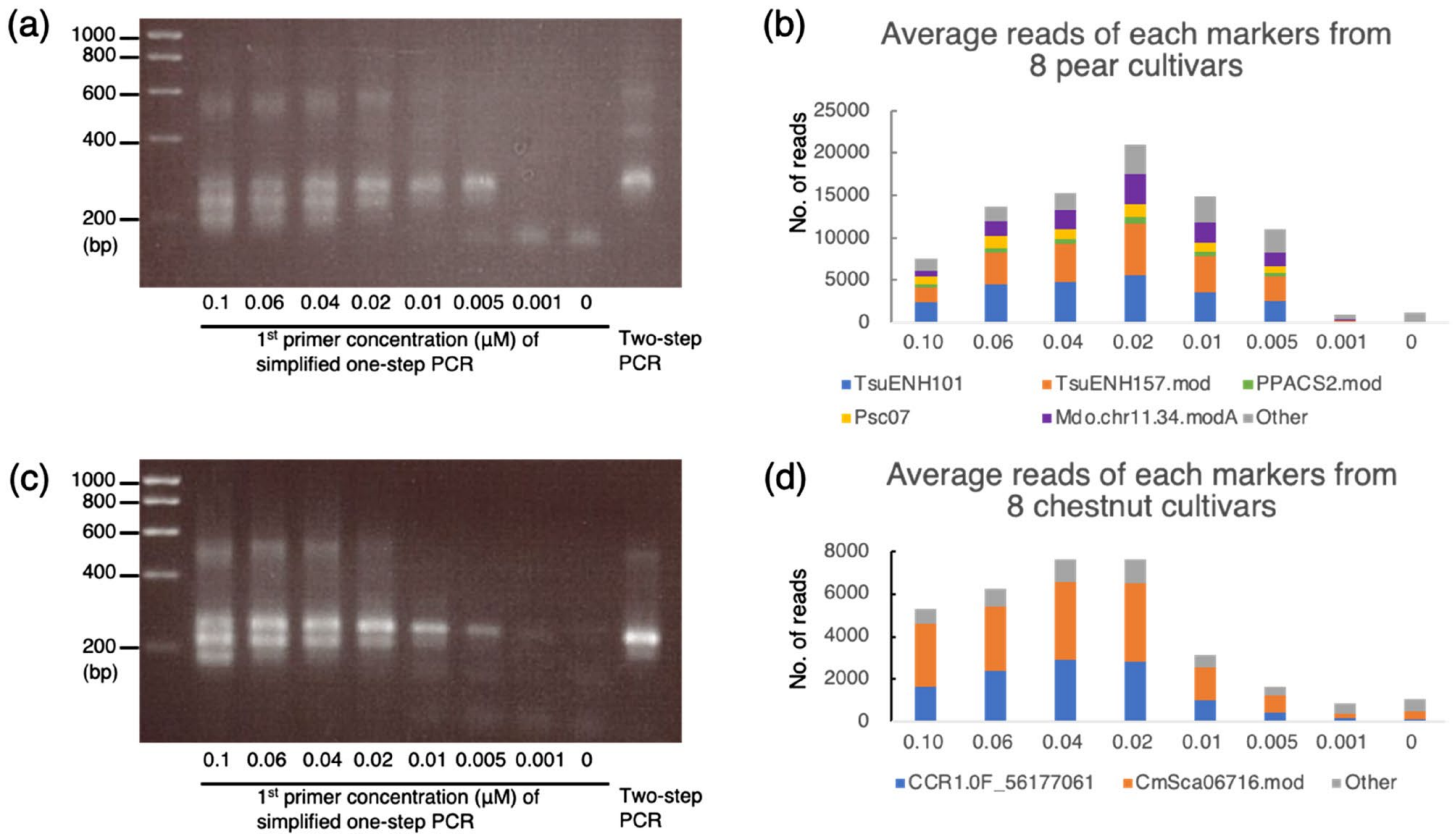
Electrophoresis of PCR products and number of reads for each marker obtained by using different primer concentrations. (**a**) Electrophoresis of PCR products created through simplified one-step PCR with various 1st-primer concentrations in pear ‘Wase Kozo’. The PCR products obtained through two-step PCR are included to indicate the target amplicon size. Five primer sets (TsuENH101, TsuENH57.mod, PPACS2.mod, Psc07 and Mdchr11.34.modA) were used in a single multiplex reaction for each sample. (**b**) Average numbers of reads of each marker from eight pear cultivars. (**c**) Electrophoresis of PCR products created through simplified one-step PCR with various 1st-primer concentrations in chestnut ‘Otomune’. Two primer sets (CCR1.0F5617761 and CmSca06716) were used in a single multiplex reaction for each sample. As in (**a**), a comparable reaction using two-step PCR is included at the far right. (**d**) Average numbers of reads of each marker from eight chestnut cultivars. Uncropped gel images for (**a**) and (**c**) are available in Supplementary Fig. S1.

To determine the optimal balance between the 1st and 2nd primer pair concentrations for simplified one-step PCR, we used a fixed final concentration of the 2nd primers (0.1 µM each) in combination with various final concentrations of the 1st primers (0, 0.001, 0.005, 0.01, 0.02, 0.04, 0.06 and 0.1 µM each) using eight cultivars each of pear and chestnut (Fig. 2a–d). Because the amplifications among the eight cultivars within a species were similar at the same 1st-primer concentrations, the amplifications from one cultivar per species using different 1st-primer concentrations were examined by agarose gel electrophoresis to confirm the intensity and distribution of the bands (Fig. 2a, c; Supplementary Fig. S1). For each cultivar, the PCR products from two-step PCR were used as a control during agarose gel electrophoresis to indicate the target band size. This analysis showed amplicons of the target size when the 1st-primer concentration was 0.01 to 0.1 µM, but the amplicons were inconsistent when the concentration was 0.005 µM, and no clear amplicons were detected when using concentrations of 0 and 0.001 µM. The amplicons of the markers used in this study were 107 to 136 bp, so the bands extended with Illumina flow-cell sequences and indexes would be 242–271 bp if amplification were successful with both the 1st and 2nd primer sets. On the other hand, if the amplicons originated only from the 1st primer pairs, which lack the flow-cell binding site and index sequences, they would range from about 174–205 bp. The overall intensities of bands in both pear and chestnut were stronger as the concentration of the 1st primer increased (Fig. 2a, c). But at 1st-primer concentrations of 0.04–0.1 µM, the frequency of short amplicons (174–205 bp), which do not carry sequences amplified by the 2nd primer set, also increased.

To assess the efficiency of amplification at different primer concentrations, we submitted an equal volume of each final reaction mixture for analysis on the Illumina MiSeq platform. The total number of sequences increased as 1st-primer concentration increased from 0 to 0.02 µM and then decreased at concentrations from 0.02 to 0.1 µM in both pear and chestnut (Fig. 2b, d), consistent with the intensities of bands around 242–271 bp viewed by agarose gel electrophoresis. The proportion of amplicons that originated from each primer pair (marker) was stable across different 1st-primer concentrations (Fig. 2b and d). Obtaining sequences amplified by both the 1st and 2nd primers (here, amplicons distributed around 242–271 bp) is crucial for NGS. On the other hand, amplified sequences that do not carry the 2nd-primer sequences (here, ~ 174–205 bp) are not useful for NGS and would add to the background. We concluded that concentrations of 0.02 µM for the 1st primers and 0.1 µM for the 2nd primers would be optimal on the basis of the numbers of reads obtained in NGS (Fig. 2b, d) and the balance between complete and incomplete amplicons (Fig. 2a, c).

### Allelic composition of diploid cultivars

**Figure 3:**
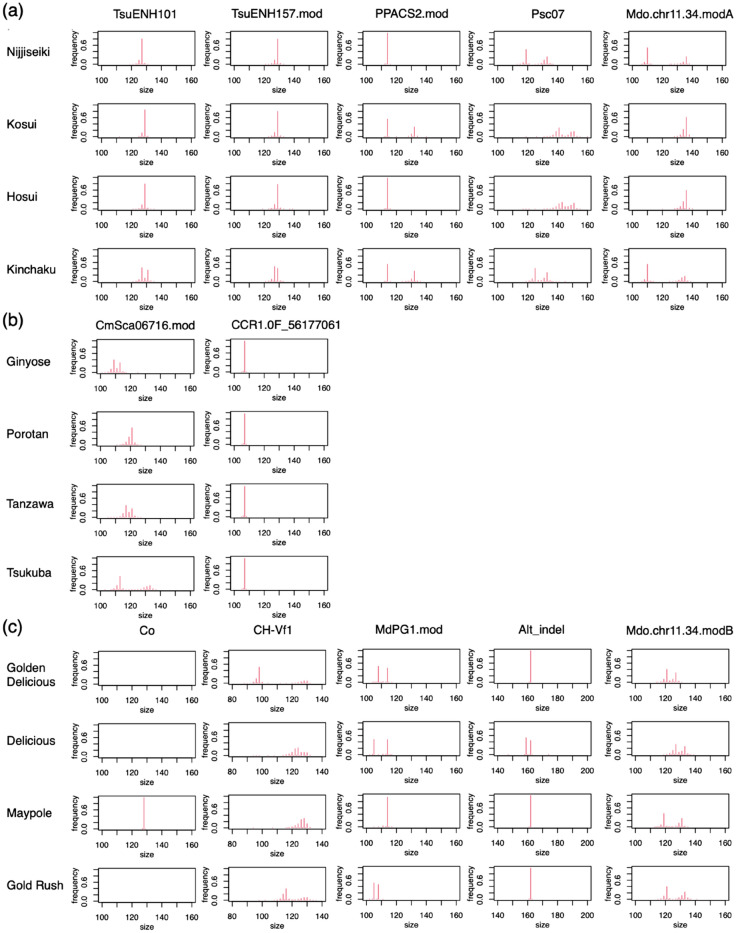
Digital electropherograms of molecular markers used in this study: electropherograms of (**a**) five molecular markers for pear, (**b**) two for chestnut and (**c**) five for apple. All of the molecular markers other than CCR1.0F_56177061 (SNP), Co (dominant marker) and Alt_indel (indel marker) are SSRs.

Using the primer concentrations determined in the above experiments, pear and chestnut cultivars were genotyped by both simplified one-step PCR (simplified AmpSeq) and two-step PCR (standard AmpSeq), and apple cultivars and segregating seedlings of pear, chestnut and apple were genotyped by simplified one-step PCR (Supplementary Tables S3–S5). The four most common alleles per locus were identified and their frequencies were calculated to estimate the genotype of each cultivar. Generally, the first and second alleles were sufficient to determine the genotypes, but the third and fourth alleles were also scored in case an allele had a high numbers of repeats, which would increase the frequency of stutter bands around that allele. Because the presence of stutter bands and differences in allele amplification made genotyping difficult, we also created digital electropherograms of the molecular marker allele frequencies obtained through the SSR-GBS pipeline (Fig. 3), which helped in assessing the extent of stutter bands. For example, marker locus Psc07 in pear has more than 24 repeats of CT in the 151-bp allele (Fig. 3a), and CH-vf1 in apple has more than 27 repeats of AG in the 128-bp allele (Fig. 3c). Those markers sometimes produced a stutter band stronger than the original band, such as the 141-bp band for Psc07 in ‘Amanogawa’ pear and the 128-bp band for CH-Vf1 in ‘Shinano Gold’ apple (Supplementary Tables S3, S5). Thus, allele calling was based on both the allele frequencies and the electropherograms. All of the genotypes determined by simplified one-step and two-step PCR in pear and chestnut were identical, suggesting that both methods have high reliability.

### Allelic composition of triploid cultivars

Four triploid cultivars (‘Santaro’, ‘Jonagold’, ‘Hokuto’ and ‘Mutsu’) from the apple cultivar collection were also genotyped, each of which would have a maximum of three alleles per locus. Although these cultivars cannot produce offspring because of their triploid chromosome constitution, they were used for allele-frequency-based genotyping to clarify whether the one-step PCR method could be applied to genotyping of polyploid species. We used markers MdPG1.mod and Alt_indel to test triploid cultivar genotyping because they produced fewer stutter bands than other markers (Table 3 and Fig. 3). For MdPG1.mod, the frequencies of the third-most-common alleles were > 0.2 in ‘Santaro’, ‘Jonagold’ and ‘Hokuto’, but < 0.03 in diploid cultivars, revealing the presence of three different alleles in each of these three triploid cultivars. ‘Mutsu’ has a frequency of 0.65 for the 108-bp allele and a frequency of 0.30 for the 114-bp allele, suggesting that it has genotype ‘108/108/114’. In contrast, ‘Indo’ (diploid), which has alleles in common with ‘Mutsu’, has a frequency of 0.49 for the 108-bp allele and of 0.45 for the 114-bp allele, suggesting that it has genotype ‘108/114’. Likewise, the Alt_indel genotype of ‘Hokuto’ is predicted to be ‘159/162/162’, because it has a frequency of 0.63 for the 162-bp allele and 0.35 for the 159-bp allele.

**Table 3:** Allele frequencies and predicted genotypes of diploid and triploid apple cultivars.

Individual	Ploidy	Trait	Marker	Total reads	Reads of target marker	Allele1	Freq1	Allele2	Freq2	Allele3	Freq3	Allele4	Freq4	Predicted genotype
Indo	Diploid	PG	MdPG1. mod	5540	1566	108	0.49	114	0.45	111	0.02	107	0.01	108/114
Delicious	Diploid			5870	1522	105	0.48	114	0.47	111	0.02	113	0.01	105/114
Fuji	Diploid			6603	1712	105	0.50	108	0.47	104	0.01	107	0.01	105/108
Gold Rush	Diploid			4873	1435	105	0.52	108	0.46	107	0.01	104	0.01	105/108
Santaro	Triploid			7284	2053	108	0.34	105	0.33	114	0.29	111	0.01	105/108/114
Jonagold	Triploid			5384	1560	108	0.33	105	0.32	114	0.31	111	0.01	105/108/114
Hokuto	Triploid			4575	968	105	0.36	108	0.33	114	0.27	107	0.01	105/108/114
Mutsu	Triploid			8565	2233	108	0.65	114	0.30	105	0.02	107	0.02	108/108/114
Indo	Diploid	Susceptibility to	Alt_indel	5540	696	159	0.57	162	0.41	174	0.01	158	0.00	159/162
Delicious	Diploid	Alternaria blotch		5870	961	159	0.53	162	0.44	174	0.01	158	0.01	159/162
Fuji	Diploid			6603	1018	162	0.99	161	0.01	NA	NA	NA	NA	162/162
Gold Rush	Diploid			4873	415	162	0.99	161	0.01	163	0.00	NA	NA	162/162
Santaro	Triploid			7284	772	162	0.99	161	0.01	NA	NA	NA	NA	162/162/162
Jonagold	Triploid			5384	520	162	0.99	161	0.01	159	0.00	NA	NA	162/162/162
Hokuto	Triploid			4575	761	162	0.63	159	0.35	161	0.01	147	0.00	159/162/162
Mutsu	Triploid			8565	976	162	0.98	161	0.02	160	0.00	NA	NA	162/162/162

Allele1 indicates the size (bp) of the most common allele, Freq1 indicates the frequency of the most common allele.

### Proportion of reads represented by each molecular marker

**Figure 4:**
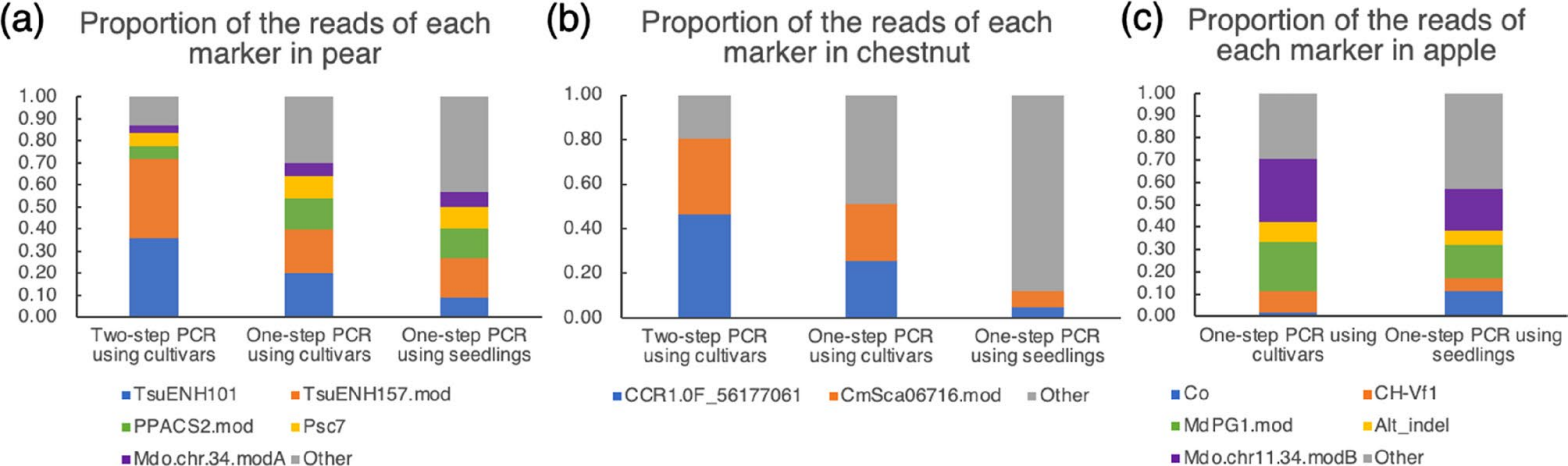
Proportion of reads represented by each marker using cultivars and seedlings of pear, chestnut and apple. (**a**) Proportion of reads represented by each marker using 59 cultivars and 96 seedlings of pear. The average numbers of reads were 8227 for two-step PCR using the cultivars, 18,683 for simplified one-step PCR using the cultivars and 17,090 for simplified one-step PCR using the seedlings. (**b**) Proportion of reads represented by each marker using 52 cultivars and 24 seedlings of chestnut. The average numbers of reads were 3657 for two-step PCR using the cultivars, 8310 for simplified one-step PCR using the cultivars and 5519 for simplified one-step PCR using the seedlings. (**c**) Proportion of reads represented by each marker using 59 cultivars and 66 seedlings in apple. The average numbers of reads were 6552 for simplified one-step PCR using the cultivars and 4022 for simplified one-step PCR using the seedlings.

Out of the total reads for each species, the average proportions represented by the sum of the marker reads were 0.87 for two-step PCR in pear cultivars, 0.70 for simplified one-step PCR in pear cultivars and 0.57 for simplified one-step PCR in pear seedlings (Fig. 4). The proportions for chestnut were 0.85 for two-step PCR in cultivars, 0.59 for simplified one-step PCR in cultivars and 0.10 for simplified one-step PCR in seedlings. In apple, those proportions were 0.71 for simplified one-step PCR in cultivars and 0.57 for simplified one-step PCR in seedlings. In both pear and chestnut, the marker reads represented a higher proportion of the total reads in two-step PCR than in simplified one-step PCR. On the other hand, the amplification bias (i.e., the variation in amplification efficiency among different markers) using two-step PCR (0.36, 0.36, 0.06, 0.06 and 0.03 for the five markers in pear; 0.46 and 0.34 for two markers in chestnut) was larger than in simplified one-step PCR (0.20, 0.19, 0.14, 0.10 and 0.05 in pear; 0.26 and 0.25 in chestnut). Because DNA samples from the seedlings were extracted by a simple method and were of only moderate quality, the proportion of marker reads was lower in seedlings than in cultivars. In particular, the sum of target marker reads was low in chestnut seedlings because of the high rate of primer dimer sequences. Nevertheless, the result of the genotyping was acceptable for practical use for MAS (Supplementary Table 4), as there were enough reads (mean 256 and 406 reads for each marker) and clear allele frequency values.

### Repeatability and correlations between simplified one-step and two-step PCR

The repeatability and correlations between simplified one-step and two-step PCR were calculated using allele frequencies of the first (i.e., most common) alleles (Table 4). The first-allele frequency values were considered to be a good indicator of repeatability because these values generally represent the most important information for estimating SSR genotypes. The frequencies of the first alleles were generally distributed around 0.8 when the genotype was homozygous and around 0.4 when the genotype was heterozygous (Supplementary Tables 3 and 5), though these values fluctuated depending on the extent of stutter bands, competition with amplification of other alleles and primer dimers. The repeatability for simplified one-step PCR was high at 0.965 to 0.996, which is sufficient for genotyping in a breeding program. The repeatability for two-step PCR was also high at 0.939 to 0.996, similar to that for simplified one-step PCR. The correlation between simplified one-step and two-step PCR ranged from 0.956 to 0.995. In summary, the allele frequency-based evaluation was repeatable for both simplified one-step and two-step PCR.

**Table 4:** Repeatability and correlation between simplified one-step and two-step PCR based on the frequency of the first (most common) allele.

Marker	Plant	Repeatability of simplified one-step PCR	Repeatability of two-step PCR	Correlation between simplified one-step and two-step PCR
TsuENH101	Pear	0.993	0.993	0.992
TsuENH157.mod		0.995	0.996	0.995
PPACS2.mod		0.996	0.993	0.994
Psc07		0.979	0.951	0.964
Mdo.chr11.34.modA		0.965	0.939	0.956
CH-Vf1	Apple	0.958		
MdPG1.mod		0.995		
Alt_indel		0.963		
Mdo.chr11.34.modB		0.982		
CmSca06716.mod	Chestnut	0.984	0.990	0.982
CCR1.0F_56177033		0.996	0.994	0.995

### Use of simplified AmpSeq to identify individuals carrying target alleles

Generally, allele calling and genotyping are conducted before selection of seedlings that carry the target genes. But this step is laborious and time consuming and is not necessary for practical MAS in a breeding program. Instead of determining genotype, we used the frequency of the target (desired) allele in each individual to determine which ones carried that allele. As an example, we summarized the results for TsuENH157.mod, PPACS2.mod and Psc07 from pear seedlings (Table 5), which are related to scab resistance, fruit-ripening day and fruit skin color, respectively. For TsuEHH157.mod, a seedling was determined to carry the resistance allele (127 bp) when the frequency of the allele in that individual was > 0.30. Likewise, for PPACS2.mod, a seedling was determined to carry the early-harvesting allele when the frequency of the 132-bp allele was > 0.20. For Psc07, which frequently produces a stutter band, the judgment criterion was lowered to 0.10; thus, when the frequency of the 151-bp allele was > 0.10, the seedlings were judged to carry the allele related to red fruit skin color. Out of 10 seedlings listed in Table 5, only seedling 7 carried the target alleles for all three markers and remained after selection.

**Table 5:** Example of marker-assisted selection through frequency of target alleles using a population derived from Tsukuba 60 and 592–021.

Individual	Trait	Marker	Total reads	Reads of target marker	Allele1	Freq1	Allele2	Freq2	Allele3	Freq3	Allele4	Freq4	Frequency of target allele	Evaluation
Tsukuba 60	Scab resistance	TsuENH 157.mod	10,597	3016	**127**	0.48	129	0.39	125	0.08	131	0.02	**0.48**	Resistant
592–021			9283	2859	129	0.45	131	0.40	127	0.09	133	0.03	0.09	Susceptible
Seedling 1			11,028	2938	129	0.80	**127**	0.13	131	0.03	125	0.02	0.13	Susceptible
Seedling 2			11,368	3574	129	0.48	131	0.36	127	0.10	133	0.02	0.10	Susceptible
Seedling 3			5786	1516	**127**	0.41	131	0.38	129	0.10	125	0.07	**0.41**	Resistant
Seedling 4			11,885	3314	**127**	0.44	131	0.37	129	0.09	125	0.06	**0.44**	Resistant
Seedling 5			13,207	3602	**127**	0.47	129	0.42	125	0.07	131	0.02	**0.47**	Resistant
Seedling 6			11,114	2857	**127**	0.47	129	0.42	125	0.08	131	0.02	**0.47**	Resistant
Seedling 7			10,381	2712	**127**	0.49	129	0.39	125	0.08	131	0.01	**0.49**	Resistant
Seedling 8			14,526	4513	129	0.49	131	0.39	127	0.08	133	0.02	0.08	Susceptible
Seedling 9			9231	2253	**127**	0.36	131	0.28	129	0.19	125	0.07	**0.36**	Resistant
Seedling 10			6214	1456	**127**	0.39	131	0.38	129	0.11	125	0.05	**0.39**	Resistant
Tsukuba 60	Fruit-ripening day	PPACS2	10,597	2025	114	0.56	**132**	0.31	130	0.08	128	0.02	**0.31**	Early harvesting
592–021			9283	2299	114	0.98	113	0.01	112	0.01	116	0.00	0.00	Late harvesting
seedling 1			11,028	2149	114	0.98	113	0.01	112	0.01	115	0.00	0.00	Late harvesting
Seedling 2			11,368	2550	114	0.57	**132**	0.28	130	0.09	113	0.02	**0.28**	Early harvesting
Seedling 3			5786	1061	114	0.98	113	0.01	112	0.01	116	0.00	0.00	Late harvesting
Seedling 4			11,885	2506	114	0.98	113	0.01	112	0.00	116	0.00	0.00	Late harvesting
Seedling 5			13,207	2675	114	0.98	113	0.01	112	0.00	116	0.00	0.00	Late harvesting
Seedling 6			11,114	2018	114	0.97	113	0.01	112	0.01	116	0.00	0.00	Late harvesting
Seedling 7			10,381	1673	114	0.59	**132**	0.28	130	0.07	134	0.02	**0.28**	Early harvesting
Seedling 8			14,526	3340	114	0.56	**132**	0.31	130	0.08	128	0.02	**0.31**	Early harvesting
Seedling 9			9231	1874	114	0.81	113	0.08	132	0.05	130	0.02	0.05	Late harvesting
Seedling 10			6214	980	114	0.93	113	0.03	132	0.02	112	0.01	0.02	Late harvesting
Tsukuba 60	Fruit skin color	Psc07	10,597	1306	141	0.29	**151**	0.17	139	0.13	149	0.12	**0.17**	Red
592–021			9283	1819	119	0.42	133	0.27	131	0.13	117	0.07	0.00	Green
Seeding 1			11,028	1726	119	0.52	141	019	139	0.11	117	0.07	0.00	Green
Seeding 2			11,368	2080	119	0.49	141	0.20	139	0.10	117	0.07	0.00	Green
Seeding 3			5786	766	133	0.38	141	0.19	131	0.13	139	0.11	0.00	Green
Seeding 4			11,885	1559	133	0.32	141	0.24	139	0.14	131	0.13	0.00	Green
Seeding 5			13,207	1784	133	0.31	141	0.25	131	0.14	139	0.12	0.00	Green
Seeding 6			11,114	1528	119	0.52	**151**	0.16	149	0.11	117	0.07	**0.16**	Red
Seeding 7			10,381	1268	133	0.32	**151**	0.17	131	0.15	149	0.12	**0.17**	Red
Seeding 8			14,526	2760	133	0.34	141	0.21	131	0.14	139	0.13	0.00	Green
Seeding 9			9231	1277	133	0.22	141	0.18	139	0.11	131	0.11	0.00	Green
Seeding 10			6214	670	119	0.48	141	0.21	139	0.10	117	0.08	0.00	Green

In the “allele” columns, marker alleles linked to the target gene are underlined and indicated in bold.

In the “Frequency of target allele” column, target allele frequencies meeting or exceeding the threshold for the presence of that allele are underlined and indicated in bold.

## Discussion

Here we developed a new method for constructing an amplicon sequencing library by using one-step PCR. This method, which we call simplified AmpSeq, reduces the time and labor needed for library construction. The amount of DNA required for our method is low (< 5 ng), because it uses a simple PCR reaction. This is an advantage for applying this method to MAS, as breeders want to conduct MAS at the juvenile stage, ideally just after germination, using a simple method. Also, we demonstrated that DNA of moderate quality could be used in this method. Generally, MAS is conducted using DNA extracted by a simple and cost-effective extraction method^21,22^; the quantity and quality of DNA used for MAS is not high but is satisfactory for use as a PCR template. For some chestnut seedlings that had low-quality DNAs extracted by very simple methods^20,21^, MAS based on allele frequency was possible, although the number of target reads was reduced (Supplementary Tables 3–5; Fig. 4b).

As an alternative to the simplified AmpSeq method developed in this study, an amplicon library can be constructed using long primer sets containing Illumina flow-cell binding regions, indexes, R1 and R2 seq primer sequences and target primer sequences. But the cost of the long primer sets would be exceedingly expensive when the number of samples is more than a thousand. Here, we divided the long primer sequences into 1st primers, which include target primer sequences and R1 and R2 seq (anchor sequences), and 2nd primers, which include Illumina flow-cell binding regions, indexes and R1 and R2 seq. This makes it easier for users to replace the target primer sequences and reduces the cost of developing target markers combined with different indexes. It is very common that the primer pairs for target traits would be updated by fine mapping of those traits^23–25^, suggesting that flexibility of the 1st primer set is very important for MAS.

The simplified AmpSeq method has several advantages over two-step PCR. In addition to reducing the procedure time and reagent cost, it produced less variation among marker sets (Fig. 4), probably owing to fewer PCR cycles. Although primer dimers and other superfluous products were more frequent in the simplified one-step PCR, they can be easily eliminated by discarding reads shorter than 80 bp. Both the simplified one-step and two-step PCR methods had high repeatability, and the difference in first-allele frequencies was negligible (Table 4). Therefore, it is reasonable to use this new simplified one-step PCR for MAS in practical breeding programs. The key to success of the simplified one-step PCR is optimal balance between the concentrations of the 1st and 2nd primer sets, which was found to be at concentrations of 0.02 µM and 0.1 µM, respectively. Because the PCR products from the 2nd primer sets were longer than those from the 1st primer sets, the amplification from the 2nd primer sets generally produced fewer PCR products. By reducing the concentration of the 1st primer sets relative to the 2nd primer sets, the depletion of 1st primer sets during the 35 cycles of PCR combined with amplification from the 2nd primer sets reduced the amount of additional amplification from the 1st primer sets. When the concentrations of the 1st primer sets were as high as 0.4× to 1.0× those of the 2nd primer sets (Fig. 2), amplification from the 1st primer sets continued and produced undesirable background from the resulting short PCR products, resulting in smaller numbers of reads detected in NGS.

Basically, any sequencing platform capable of producing paired-end reads can accommodate libraries constructed using our method. However, an inherent issue called “index hopping” arises in which misassignments occur across multiple samples sequenced in the same lane^26–28^. The rate of index hopping varies depending on the type of sequencer. The sequencers that have a nonpatterned flow cell, including MiSeq and NextSeq, showed lower levels of index hopping than those that have a patterned flow cell, including Hiseq X and NovaSeq^29^. Thus, we used MiSeq for demonstrating MAS; in addition to having a nonpatterned flow cell, this platform has several types of reagent kits including nano, micro and standard configurations, depending on the number of sequence reads (2–30 million). The number of paired-end reads produced by MiSeq Reagent kit V2 is 24–30 million (300 cycle, Illumina), which is equivalent to 12–15 million for single reads. As the numbers of reads per genotype by NGS for SSRs are ideally 100–1000^30–32^, simplified AmpSeq with several markers can be conducted with 5000 reads per marker per sample, which means that more than a thousand samples can be covered by the single MiSeq run. When the target markers are SNPs or indels, the number of reads per sample can be lowered, as the basic pipeline for detecting SNPs is based on 4× to 20× coverage^12,16,33^. For example, the annual cost for simplified AmpSeq-based MAS in the pear breeding program at the Institute of Fruit Tree and Tea Science is less than US$2000 for 1500–2000 individuals, including US$1200 for the single MiSeq run, US$200 for PCR reagents, US$200 for plastic plates, plastic tips and other reagents for library construction. Despite the expense, simplified AmpSeq-based MAS is cost-effective because it reduces the time and labor needed for evaluation. The method helps breeders to select target genotypes and gives genetic information about individuals, such as contamination from unintended cross-pollination, by using several important marker sets.

The use of simplified AmpSeq for MAS has advantages in expandability and versatility. Using target allele frequency as the selection criterion, semi-automated evaluation can be implemented, reducing the labor required compared with genotyping by visually scoring target bands after electrophoresis. Further, we demonstrated by using triploid apple cultivars that this method can detect and count duplicated alleles in polyploid species (Table 3). Because the method has high repeatability for the frequency of the first allele (Table 4), allele frequencies can be used to estimate the numbers and types of alleles in individuals from polyploid species. Furthermore, the method is applicable to many variant types, including SSRs, SNPs and indels, as well as combinations of those markers. Although SNPs, which are the most widely used markers, generally have only two alleles per locus, important genes may have more than two alleles that affect the target trait, sometimes with different levels of effect. For example, the markers associated with fruit skin color in pear and flesh mealiness in apple in this study^34,35^ each have at least three types of alleles in cultivar collections. In pear, HAP1 has a strong dominant effect on cork layer formation, HAP2 and HAP3 have a weaker dominant effect, and HAP4–HAP6 are recessive and have no effect on cork layer formation. In this case, using SSR markers that detect multiple alleles is efficient and rational for selection. Also, in the amplicon of CCR1.0F_56177061 of chestnut, a SNP was identified at ChrF:56177075, a position different from the target variant position (ChrF:56177061). In this case, haplotype-based evaluation using both variants can be applied in MAS, which may enhance selection for this trait in breeding programs. By introducing the simplified AmpSeq-based MAS, breeders will be able to design the kinds of markers that are most suitable for selection in their breeding programs.

In conclusion, we developed a simplified AmpSeq library construction method using one-step PCR that can be applied to MAS. By using pear, chestnut and apple, we demonstrated that it can be used for practical selection with high accuracy and repeatability. We also demonstrated that semi-automated evaluation can be implemented using target allele frequency as the selection criterion. By implementing simplified AmpSeq, breeders can reduce the time and labor needed for genotyping, which previously required visual detection of bands in agarose gel or fragments from a capillary sequencer, and the risk of human error. As it enables flexibility in designing primer sets and targeting any kind of sequence-based variant, this method is an option not only for the species demonstrated here, but also for breeding programs in other species.

## Materials and methods

### Plant materials, DNA extraction and molecular markers

As plant materials, 59 cultivars and 96 seedlings of Japanese pear, 46 cultivars and 24 seedlings of Japanese chestnut, and 52 cultivars and 66 seedlings of apple were used (Table 1, Supplementary Table S1). Each cultivar was represented by a single tree. Genomic DNA was extracted from young leaves of the cultivar collections with a DNeasy Plant Mini Kit (Qiagen, Germany). To determine the DNA quality needed for practical use of simplified AmpSeq-based MAS, genomic DNA was extracted from seedlings using simple and rapid DNA extraction methods. For pear, DNA was extracted from 5 mg of young leaves using a NucleoMag Plant kit (Macherey–Nagel, Germany) according to the manufacturer’s instructions, except that the amounts of tissue and reagents used were each one-quarter of the specified amounts. For chestnut, DNA was extracted from 5 mg of cotyledons using a simple method^23,36^. For apple, TPS buffer (100 mM Tris·HCl, 1 M KCl, 10 mM EDTA, pH 8.0) was used for DNA extraction with a simple isopropanol washing step^37^. Leaf sections (3– 4 cm) from each seedling were disrupted in 300 µL TPS buffer in a Multi-beads Shocker. After centrifugation, DNA was precipitated by addition of 2-propanol, washed with 70% ethanol and dissolved in 50 µL 0.1 × TE buffer (1 mM Tris·HCl, 0.1 mM EDTA, pH 8.0).

The databases for MAS were created using five molecular markers associated with disease resistance^38,39^, fruit-ripening day^40^ and fruit skin color^34^ in Japanese pear; two associated with pellicle peelability in Japanese chestnut^23,41^ and five related to disease resistance^42–44^, columnar growth type^24^ and flesh mealiness^35,45^ in apple (Table 2, Supplementary Table S2). To promote stable amplification in multiplex PCR, some of the primer pairs were re-designed to shorten the length of the product to less than 150 bp against the reference genomes of ‘Nijisseiki’ pear, ‘Ginyose’ chestnut and ‘Golden Delicious’ doubled haploid 13 (GDDH13) apple^46–48^.

### Constructing libraries for simplified AmpSeq

To construct a library for AmpSeq , a two-step PCR method based on Nishio et al.^31^ and a newly developed simplified one-step PCR method were used. Both methods require the primers to contain Illumina flow-cell binding sites, 8-bp indexes and sequences for determining the insert sequences on both sides of the amplicons (Fig. 1).

In two-step PCR, the 1st PCR was performed using target-specific primers extended with the forward R1 seq primer (5′-ACACTCTTTCCCTACACGACGCTCTTCCGATCT-3′) and the reverse R2 seq primer (5′-GTGACTGGAGTTCAGACGTGTGCTCTTCCGATCT-3′). The five (1st) primer sets for pear, two for chestnut and five for apple were used in a single multiplex reaction for each sample (i.e., cultivar or seedling) of the corresponding species. PCR amplification was performed in 10 µL containing 5 µL of 2 × Green GoTaq G2 Hot Start Master Mix (0.4 mM each dNTP, Taq DNA polymerase and 4 mM MgCl_2_, pH 8.5; Promega, Madison, WI, USA), 0.2 µL of the 1st primer set (10 µM), 3.8 µL H_2_O and 1 µL of genomic DNA (2.5 ng/µL). Amplification was performed in an initial denaturation of 94 °C for 5 min; 25 cycles of 94 °C for 30 s, 60 °C for 1 min and 72 °C for 30 s; and 72 °C for a final extension of 10 min. The 2nd PCR for each sample was performed using a pair of long primers as the 2nd primer set, with the first primer containing the P5 sequence, an 8-bp index and the R1 seq primer (5′-AATGATACGGCGACCACCGAGATCTACAC[Index]ACACTCTTTCCCTACACGACG-3′) and the second containing the P7 sequence, a different 8-bp index and the R2 seq primer (5′-CAAGCAGAAGACGGCATACGAGAT[Index]GTGACTGGAGTTCAGACGTGT-3′). A unique combination of indexes was used for each sample and PCR method. The P5 and P7 sequences produce the Illumina flow-cell binding sites, and the two different 8-bp indexes per sample were used for demultiplexing. PCR amplification was performed in 10 µL containing 5 µL of 2× Green GoTaq G2 Hot Start Master Mix, 1 µL of the 2nd primer set (1 µM), 3 µL H_2_O and 1 µL of the 1st PCR products. PCR reactions used an initial denaturation of 94 °C for 5 min; 15 cycles of 94 °C for 30 s, 60 °C for 1 min and 72 °C for 30 s; and 72 °C for a final extension of 10 min.

In simplified one-step PCR, PCR amplification was performed in 10 µL containing 5 µL of 2 × Green GoTaq G2 Hot Start Master Mix, 0.2 µL of the 1st primer set, 1 µL of the 2nd primer set (1 µM), 2.8 µL H_2_O and 1 µL of genomic DNA (2.5 ng/µL). To clarify the optimal primer concentration for simplified one-step PCR, we used initial concentrations of the 1st primer set from 0 to 5 μM to obtain final concentrations of 0, 0.001, 0.005, 0.01, 0.02, 0.04, 0.06 or 0.1 µM, which were tested using eight cultivars each of pear and chestnut (Supplementary Table S1). The 2nd-primer concentrations were set at 0.1 µM because a primer concentration of 0.1 µM to 1.0 µM was recommended in the manual for GoTaq G2 Hot Start Master Mix and a relatively low primer concentration (0.2 µM) was recommended in some multiplex PCR kits. PCR reactions used an initial denaturation of 94 °C for 5 min; 35 cycles of 94 °C for 30 s, 60 °C for 1 min and 72 °C for 30 s; and 72 °C for a final extension of 10 min.

For pear and chestnut cultivars, the two-step and simplified one-step PCR experiments were done twice for each cultivar collection to test repeatability. For apple cultivars, only simplified one-step PCR was done. Simplified one-step PCR was also applied to seedlings of pear, chestnut and apple to check whether the method was applicable for practical MAS. In total, three libraries for NGS were constructed from the PCR products: one from cultivars and seedlings of pear and chestnut that included 614 samples, one from apple cultivars that included 208 samples and one from apple seedlings that included 132 samples. In each library, all of the 2nd PCR products of two-step PCR and/or the PCR products of simplified one-step PCR were mixed equally by volume in a single tube and purified using AMPure XP beads (Beckman Coulter, Inc., Bree, CA, USA) following the Agencourt AMPure XP PCR Purification protocol. The library concentrations were quantified with a Qubit 3.0 fluorometer (Thermo Fisher Scientific) and Qubit dsDNA BR assay kit (Thermo Fisher Scientific). The libraries were sequenced by PE 150-bp sequencing on an Illumina MiSeq platform (Illumina, Inc., San Diego, CA, USA).

### Data analyses for simplified AmpSeq

Data were analyzed by means of the SSR-GBS pipeline^31^ with a slight modification. The reads from Illumina MiSeq were demultiplexed to each cultivar or seedling and PCR method on the basis of the index sequences, and paired fastq files of each cultivar were obtained. The fastq files were trimmed of adapter sequences and low-quality bases in Trimmomatic v. 0.39 software^49^. The paired fastq files were merged in flash2 software^50^ with parameters “-M 150 -X 0.05—allow-outies”. The merged reads were then demultiplexed on the basis of target primer sequences. For indels and SSRs, the number of reads from each allele (defined by allele length) in each cultivar was counted by a basic Linux command using a custom script (Supplementary file 1). The four most common alleles were extracted and their allele frequencies were calculated. For SNP marker CCR1.0F_56177061, haplotype frequencies were calculated because a second SNP was identified at ChrF:56177075, a position different from the target variant position (ChrF:56177061). The three major haplotypes among the 46 chestnut cultivars were designated as HAP1, HAP2 and HAP3. The merged reads were aligned and stacked by sequence, and the allele frequency of the two most common haplotypes was calculated for each individual by using a custom script (Supplementary file 2).

Cultivars were genotyped using the allele frequency data and digital electropherograms created in R v. 4.2.2 software (R Development Core Team). The allele frequency of the target band associated with each trait was calculated to determine which individuals carried the target allele. Generally, the frequencies of target alleles associated with each trait provided sufficient information for genotyping, but the frequencies of other alleles helped in detecting the presence of stutter bands and the accuracy of the called genotype.

## Supplementary Information

Supplementary Figure S1.

Supplementary Tables.

Supplementary Information.

## Data Availability

The datasets supporting the conclusions of this article are included within the article and its supplementary information files. Sequence reads are available from the Sequence Read Archive (DRA) of DNA Data Bank of Japan (DDBJ) under the accession numbers of DRA16007, DRA16008 and DRA 16009.
