# Supplementary material for: Rapid and easy construction of a simplified amplicon sequencing (simplified AmpSeq) library for marker-assisted selection
Source: Sci Rep. 2023 Jun 29;13:10575. doi: 10.1038/s41598-023-37522-1 (PMC10310812; doi:10.1038/s41598-023-37522-1)
Supplement: Supplementary file 1 — Supplementary Figure S1. [file 41598_2023_37522_MOESM1_ESM.pptx]

## Slide 1
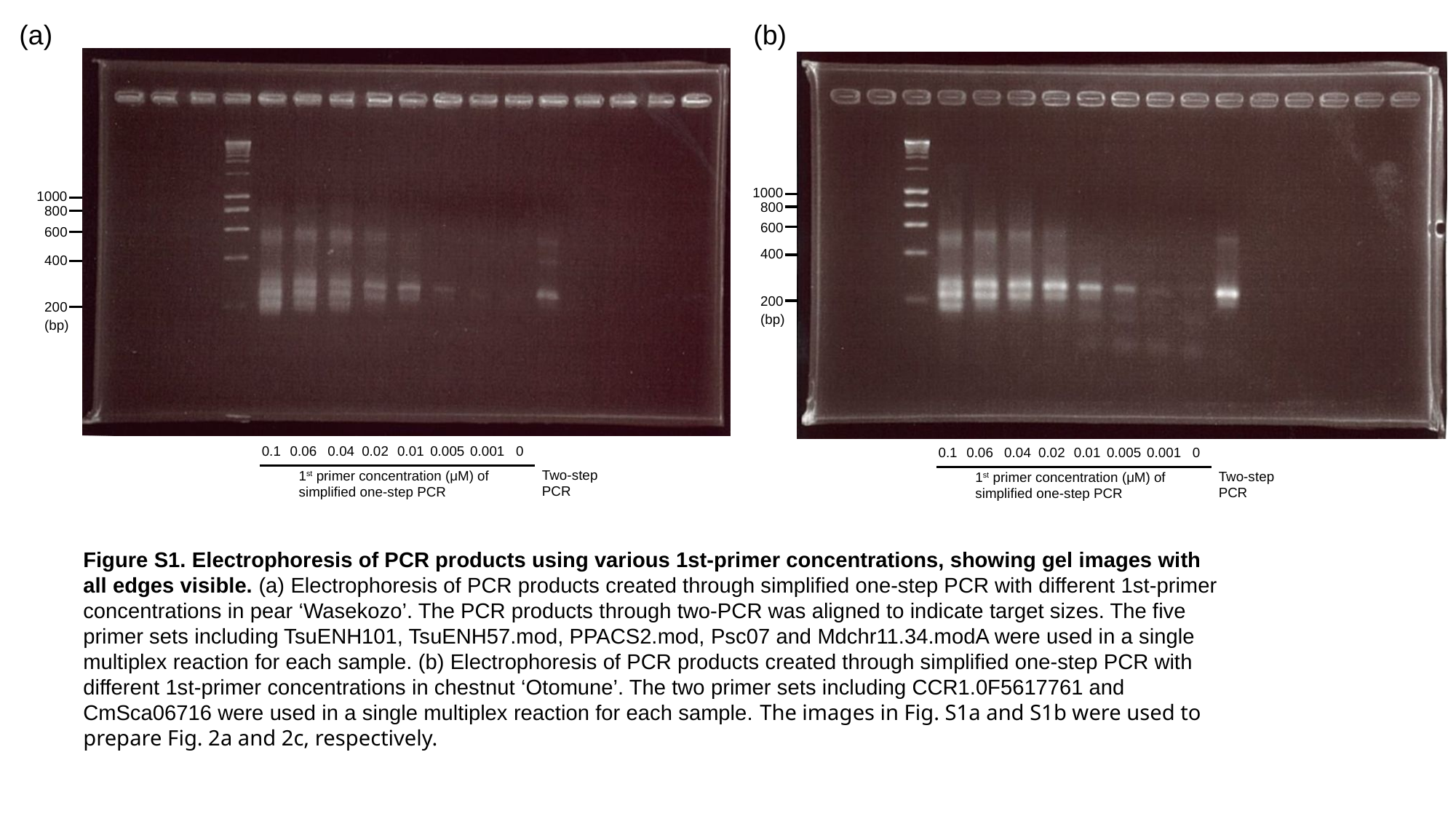

(b)
(a)
1000
1000
800
800
600
600
400
400
200
200
(bp)
(bp)
0.1
0.06
0.04
0.02
0.01
0.005
0.001
0
0.1
0.06
0.04
0.02
0.01
0.005
0.001
0
Two-step
PCR
1st primer concentration (μM) of
simplified one-step PCR
Two-step
PCR
1st primer concentration (μM) of
simplified one-step PCR
Figure S1. Electrophoresis of PCR products using various 1st-primer concentrations, showing gel images with all edges visible. (a) Electrophoresis of PCR products created through simplified one-step PCR with different 1st-primer concentrations in pear ‘Wasekozo’. The PCR products through two-PCR was aligned to indicate target sizes. The five primer sets including TsuENH101, TsuENH57.mod, PPACS2.mod, Psc07 and Mdchr11.34.modA were used in a single multiplex reaction for each sample. (b) Electrophoresis of PCR products created through simplified one-step PCR with different 1st-primer concentrations in chestnut ‘Otomune’. The two primer sets including CCR1.0F5617761 and CmSca06716 were used in a single multiplex reaction for each sample. The images in Fig. S1a and S1b were used to prepare Fig. 2a and 2c, respectively.
