# Supplementary material for: Rapid and easy construction of a simplified amplicon sequencing (simplified AmpSeq) library for marker-assisted selection
Source: Sci Rep. 2023 Jun 29;13:10575. doi: 10.1038/s41598-023-37522-1 (PMC10310812; doi:10.1038/s41598-023-37522-1)
Supplement: Supplementary file 3 — Supplementary Information. [file 41598_2023_37522_MOESM3_ESM.pdf]

Title:

Rapid and easy construction of a simplified amplicon sequencing (simplified AmpSeq) library for marker-assisted selection

Authors:

Sogo Nishio[1], Shigeki Moriya[2], Miyuki Kunihisa[1], Yukie Takeuchi[1], Atsushi Imai[1] and Norio Takada[1]

Authors' addresses:

[1] Institute of Fruit Tree and Tea Science, NARO, 2-1 Fujimoto, Tsukuba, Ibaraki, Japan

[2] Institute of Fruit Tree and Tea Science, NARO, Morioka, Iwate 020-0123, Japan

***Corresponding author:**

Sogo Nishio

Institute of Fruit Tree and Tea Science, NARO, 2-1 Fujimoto, Tsukuba, Ibaraki 305-8605, Japan; Tel: +81-29-838-6464

nishios@affrc.go.jp

```
#Script1.sh
# !/bin/sh
Basedir=/Volumes/SSRGBS/2022Miseq/MAS_Chestnutpaper/
Readdir=$Basedir"raw/";# Putting the fastq.gz files in this folder
Cleandir=$Basedir"0.Clean/";
Samplelist=$Basedir"script/list_geno";
# "list_geno" includes the IDs of varieties corresponding to the filenames (XXX.fastq.gz)
Primer=$Basedir"script/list_primer";
# "list_primer" includes marker name, forward primer sequence, reverse primer sequence in space-separated format.
Combinedir=$Basedir"1.Combine/";
Demultidir=$Basedir"2.Demulti/";
Resultdir=$Basedir"3.Result/";
listNo=`cat $Primer|wc -l`;
Summarydir=$Basedir"4.Summary/"
# Cleaning the pairs of fastqs files obtained from NGS
mkdir $Cleandir $Combinedir $Demultidir $Resultdir $Summarydir
ls $Readdir
for i in $(cat $Samplelist);
 do
  trimmomatic \
  PE                    \
  -threads 4            \
  -phred33              \
  -trimlog $Cleandir"log.txt"       \
  $Readdir"$i"_1.fastq.gz \
  $Readdir"$i"_2.fastq.gz \
  $Cleandir"clean_trim_"$i"_1.fastq.gz" \
  $Cleandir"clean_unpaired_"$i"_1.fastq.gz" \
  $Cleandir"clean_trim_"$i"_2.fastq.gz" \
  $Cleandir"clean_unpaired_"$i"_2.fastq.gz" \
  ILLUMINACLIP:$Basedir"script/adapter.fa:2:30:10" \
  SLIDINGWINDOW:4:15 \
  MINLEN:60
  rm  $Cleandir"clean_unpaired_"$i"_1.fastq.gz" $Cleandir"clean_unpaired_"$i"_2.fastq.gz"
done

# Combining the pairs of cleaned fastqs files
mkdir $Combinedir;
for i in $(cat $Samplelist);
 do
  flash2 -M 150 -x 0.05 --allow-outies \
  $Cleandir"clean_trim_"$i"_1.fastq.gz" $Cleandir"clean_trim_"$i"_2.fastq.gz";
  cat out.extendedFrags.fastq| gzip -c > $Combinedir"$i"_comb.fastq.gz;
done
rm out*

for i in `seq $listNo`;
 do
# adjusting format of the primers
Marker=$(sed -n "$i"p $Primer | awk '{print $1}')
DemultiMarkerdir=$Demultidir$Marker/;
Primer_F=$(sed -n "$i"p $Primer | awk '{print $2}')
```

```
Primer_R=$(sed -n "$i"p $Primer | awk '{print $3}')
echo $Marker $Primer_F $Primer_R
Primer_F_sort=`echo $Primer_F | sed -e 's/^.//' | sed -e "y/atgc/ATGC/"`
Primer_R_sort=`echo $Primer_R | sed -e 's/^.//' | rev | sed -e "y/atgc/ATGC/"|sed -e "y/ATGC/TACG/"`
echo $Primer_F_sort $Primer_R_sort

# demultiplexing based on the primer sequences
mkdir $DemultiMarkerdir
for j in $(cat $Samplelist);
 do
 gzcat  $Combinedir"$j"_comb.fastq.gz | grep -i -B 1 -A 2 $Primer_F_sort \
|grep -i -B 1 -A 2 $Primer_R_sort|grep -v "^--$"  \
|gzip > $DemultiMarkerdir"$Marker_$j".fastq.gz
done;
done

# Creating the folders to put files for calculation
for i in `seq $listNo`;
 do
Marker=`cat $Primer|awk '{print $1}'|sed -n "$i"p`;
Marker_read=`echo $Marker"_read"`
DemultiMarkerdir=$Demultidir$Marker/;
ResultMarkerdir=$Resultdir$Marker/;
mkdir $ResultMarkerdir

# Retrieving numbers of reads, lengths of alleles and allele frequencies
for j in $(ls $DemultiMarkerdir|tr " " "¥n" |sed 's%.fastq.gz%%g');
 do
# Counting the numbers of alleles
  gzcat $DemultiMarkerdir"$j".fastq.gz|awk 'NR%4 == 2'|awk 'length($0)>80'|awk 'length($0)<500' \
  |awk ' {lengths[length($0)]++} END {for (l in lengths) {print NR, l, lengths[l],lengths[l]/NR }}' \
  |sort -k3 -n -r > $ResultMarkerdir"$j"_$Marker.count;
done;

for j in $(ls $DemultiMarkerdir|tr " " "¥n" |sed 's%.fastq.gz%%g');
 do
# Obtaining output information for the first four alleles
  total_num=`gzcat $Readdir"$j"_1.fastq.gz |awk 'NR%4 == 2'|wc -l`
  comb_num=`gzcat $Combinedir"$j"_comb.fastq.gz |awk 'NR%4 == 2'|wc -l`
  comb100_num=`gzcat $Combinedir"$j"_comb.fastq.gz |awk 'NR%4 == 2'|awk 'length($0)>100'|wc -l`
  num=`cat $ResultMarkerdir"$j"_$Marker.count | awk 'NR==1 {print $1}'`
  list=`sed -n '1,4p' $ResultMarkerdir"$j"_$Marker.count | awk '{print $2, $4}' | tr '\n' ' '`
  echo $j"_"$Marker".count" $total_num $comb_num $comb100_num $num $list\
  |sed 's/$/ NA NA NA NA NA NA NA NA NA/g'|cut -f 1-13 -d ' '
done |sed "1s/^/variety reads comb_reads comb100_reads $Marker_read rep1 freq1 rep2 freq2 rep3 freq3 rep4 freq4#/g"\
|tr '#' '\n' > $Summarydir"/summary"$Marker".txt";
done;
```

```
#Script2.sh
# !/bin/sh
Basedir=/Volumes/SSRGBS/2022Miseq/MAS_Chestnutpaper/
Readdir=$Basedir"raw/";# Putting the fastq.gz files in this folder
Cleandir=$Basedir"0.Clean/";
Samplelist=$Basedir"script/list_geno";
# "list_geno" includes the IDs of varieties corresponding to the filenames (XXX.fastq.gz)
Primer=$Basedir"script/list_primer";
# "list_primer" includes marker name, forward primer sequence, reverse primer sequence in space-separated format.
Combinedir=$Basedir"1.Combine/";
Demultidir=$Basedir"2.Demulti/";
Resultdir=$Basedir"3.Result/";
listNo=`cat $Primer|wc -l`;
Summarydir=$Basedir"4.Summary/"

Primer_F=`echo tgcttgtgcttcttgaaagg | sed -e "y/atgc/ATGC/"`
Primer_R_sort=`echo ccggaatcaaagagcagaga | sed -e 's/^.//' | rev | sed -e "y/atgc/ATGC/"| sed -e "y/ATGC/TACG/"`

for j in $(cat $Samplelist);
 do
   CombNum=`gzcat $Combinedir"$j"_comb.fastq.gz |awk 'NR%4 == 2'|wc -l`
   MarkerNum=`gzcat $Combinedir"$j"_comb.fastq.gz |awk 'NR%4 == 2'|awk 'length($0)>80'|awk 'length($0)<180' \
   |grep -i $Primer_F |grep -i $Primer_R_sort|wc -l`
   hap=`gzcat $Combinedir"$j"_comb.fastq.gz |awk 'NR%4 == 2'|awk 'length($0)>80'|awk 'length($0)<180' \
   |grep -i $Primer_F |grep -i $Primer_R_sort \
   |sort -n|uniq -c|sort -n -r|head -n 4|tr '\n' '\t'`
   echo $j $CombNum $MarkerNum $hap|awk '{print $1,$2,$3,$5,$4/$3,$7,$6/$3}'
done |sed '1s/^/variety comb_reads target_reads hap1 freq1 hap2 freq2\n/g'> $Summarydir"SEQ_CCR1.0F_56177061.txt";

cat $Summarydir"SEQ_CCR1.0F_56177061.txt" \
|sed 's/TGCTTGTGCTTCTTGAAAGGATGGAACTTTGTGTTTTTTTAATGATTATTTGATGGAGAGAGATGGGGTTGATGGAGGATTGGAGTTTCTCTGCTCTTTGATTCCGG/HAP1/g' \
|sed 's/TGCTTGTGCTTCTTGAAAGGATGGAACTTCGTGTTTTTTTAATGATTATTTGATGGAGAGAGATGGGGTTGATGGAGGATTGGAGTTTCTCTGCTCTTTGATTCCGG/HAP3/g' \
|sed 's/TGCTTGTGCTTCTTGAAAGGATGGAACTTTGTGTTTTTTTAATAATTATTTGATGGAGAGAGATGGGGTTGATGGAGGATTGGAGTTTCTCTGCTCTTTGATTCCGG/HAP2/g' \
|awk '{
    sub("'TGCTTG'.*.", "'NA'");
    print $0;
}'> $Summarydir"HAP_CCR1.0F_56177061.txt"
```
